# COVID-19 and orthopaedic surgeons: the Indian scenario

**DOI:** 10.1177/0049475520921616

**Published:** 2020-04-21

**Authors:** Vijay K Jain, Raju Vaishya

**Affiliations:** 1 Associate Professor, Department of Orthopaedics, Atal Bihari Vajpayee Institute of Medical Sciences, Dr. Ram Manohar Lohia Hospital, New Delhi, India; 2 Senior Consultant, Department of Orthopaedic, Joint Replacement and Arthroscopic Surgery, Indraprastha Apollo Hospitals, New Delhi, India

**Keywords:** COVID-19, coronavirus disease, orthopaedics, trauma, surgery

## Abstract

The emergence of COVID-19 has impacted orthopaedic surgery worldwide. India, with its large population and limited health resources, will be overwrought over the coming days due to the number of cases of critically ill patients with COVID-19. It is important to understand the challenges for orthopaedic (and other) surgeons in India when dealing with patients during the COVID-19 pandemic. This article highlights the challenges in the triaging of patients, care in dealing with a patient with COVID-19 in orthopaedic surgery, and the effects on academics and research activities; it also suggests immediate measures and recommendations that also apply to other specialties.

## Background

The emergence of Coronavirus Disease 2019 (COVID-19) has impacted orthopaedic surgery globally. Following the recommendations of the Ministry of Health and Family Welfare, Government of India, all major private and public hospitals have been advised to delay or postpone elective surgeries during the COVID-19 outbreak. To overcome this situation, the Prime Minister of India has urged people to stay at home and practise social distancing until 3 May, and has imposed a complete lockdown on the whole nation. This will hopefully decrease the rise in COVID-19 cases. If the situation gets to the same level as that of Italy, Iran or France, we can predict that all elective orthopaedic practices will be cancelled. It is anticipated that there may later be a surge of orthopaedic patients, delayed in their treatment, with significantly increased morbidity. The slack that exists in countries richer in resources does not exist in India. This may well mean that those patients having elective surgery delayed or cancelled will, in fact, never get such surgery done.

In India, orthopaedic outpatient and emergency departments in public and private hospitals are often flooded with patients, requiring emergency and non-emergency care. This situation has become more challenging for healthcare professionals since the COVID-19 outbreak. There are approximately 20,000 orthopaedic surgeons available to cater to a population of approximately 1.25 billion, i.e. one surgeon for every 62,500 people.^1^ The number of Intensive Care Unit (ICU) beds available is also disproportionately low in Indian hospitals, and obtaining such a bed for critically ill patients is difficult even for routine orthopaedic surgery. Most patients requiring elective orthopaedic services are elderly and may require ICU back-up.

The complete lockdown may reduce the number of road traffic incidents, but it is by no means proven that this will substantially reduce the need for emergency orthopaedic surgery.

## Challenges

During the coronavirus pandemic, orthopaedic surgeons and patients will have difficult choices for a wide variety of injuries and urgent orthopaedic conditions.^2,3^ Public health experts agree on the following urgent measures:

- Thoughtfully review all their scheduled elective procedures with a plan to minimise, postpone or cancel them;
- Instantaneously minimise the use of essential items needed to care for critically ill patients, including, but not limited to, ICU beds, personal protective equipment (PPE) and ventilators.

## Problems

In the current scenario, we do not know the exact status of COVID-19 in our population; therefore, performing elective or any kind of surgery without work-up of COVID-19 in a particular patient may involve risk to the whole operation theatre staff, resident doctors and faculty. We have to adopt the ‘HIV model’ for every emergency case during the COVID pandemic. All patients, unless proven otherwise, should be assumed to be positive and orthopaedic surgeons must take appropriate precautions. Contact of surgeons or anaesthetists with known COVID-19 patients will already force them to quarantine themselves voluntarily from family and friends, and so further add to a shortage of workforce. However, procuring a COVID-19 test in every patient will not be possible due to its high cost and unavailability of detection kits universally. Furthermore, with a false-negative rate of 30%, its reliability is not optimal and represents only the situation present at the moment of the test; it is not necessarily the same the day after. In addition, it must be considered that the longer the operating time, the greater the contact time between surgeon, staff and patient for any infection.

Hospital resources such as disposables, ICU beds and other equipment need to be set aside for expected COVID-19 patients. Furthermore, the existence of COVID-19 needs to be tested in blood donors, which inevitably will lead to blood product shortages due to the inadequacy of testing resources and a decline in community blood donation drives.

Therefore, for these patients, extra precautions should be taken. It is known that anaesthetists are considered particularly at risk when intubating.^4^ Full protection gear is advised, including a N95 mask with a powered air-purifying respirator with a hood covering the head and neck area, if possible. Operating theatres with negative pressure ventilation facility are recommended.

It must not be forgotten that many orthopaedic procedures generate aerosol particles, especially during pulse lavage, the use of powered instruments (a drill, saw or burr) and intramedullary reaming. Aerosols and droplets form due to the mechanical disruption of blood or other body fluids during these procedures. Pathogens surviving within these particles of < 5 micron suspended in the air may be inhaled and so propagate infection.^5^ Surgeons need full protection if high-speed devices are to be used, but could enter the operating room after intubation and successful air turnover with simple face masks and visors, if not.

The infographic from the Centers for Disease Control and Prevention on the best facial hairstyles suited to N95 respirator masks, which are intended to help shield from airborne particles, should be followed.^6,7^

Neglected orthopaedic trauma in India is already a huge problem due to delayed presentation, misdiagnosis, unjustified prolonged and unsuccessful conservative treatment, and unavailability of treatment facilities. In addition, patients treated primarily by non-specialist doctors, quacks, osteopaths or operated under suboptimal theatre conditions with poor-quality implants also contribute to this burden.^8^ This is likely to increase with COVID-19, especially with further difficulties in reaching hospital.

Further, patients tend to avoid hospitals for fear of becoming infected with COVID-19 there. The conversion of trauma care centres to COVID-19 hospitals, the closing of outpatient and routine operation theatres, lack of available anaesthesia facility and staff (diverted to ICUs), and reallocation of surgical manpower to COVID-19 wards and screening centres are other contributing factors.

## Alternative management

Conservative treatment of fractures must not be seen as ‘out of fashion’ and should be adopted wherever feasible, particularly for unreduced dislocations, septic arthritis, traumatic amputation, crush injuries, compartment syndrome, cauda equina syndrome, multiple long bone fractures and compound injuries. Temporary measures such as steroid injections should be used where appropriate to postpone interventions.^9^ Likewise, imaging that is not absolutely necessary should be avoided. Enabling family members to practise physiotherapy is key. Use of removable splints or casts also needs to be promoted.

Importantly, elective patients may also have asymptomatic COVID-19 infection, which potentially increases their mortality. Similarly, emergency patients may also have synchronous COVID-19 infection, which will increase their mortality risk for general anaesthesia; hence, a non-operative management should always be favoured over surgical intervention.

However, we need to give an excellent standard care of treatment to each patient rather than refuse them in fear of COVID-19 infection.

Nonetheless, early discharge from hospital, where feasible, must be practised.

### Care for COVID-19 patients in surgery

Minimising the number of staff in the operating theatre is mandatory. A dedicated team comprising a senior member and two senior residents, sufficient for any case, has to be established. In case of need, the help of a dedicated nurse or paramedic may be added during the surgical procedure. This team will be responsible for reviewing and operating on suspected or confirmed cases and should be kept segregated from the rest of the department to minimise the risk of cross-contamination. Alternate teams can also be created, if required, in case of heavy load of patients and/or to replace a team inadvertently exposed to an infected patient.

Extra scheduling of operating time may, paradoxically, be needed to account for special COVID-19 measures to be put in place.

Guidelines published by the Indian Orthopaedic Association should be followed by every orthopaedic surgeon across the country.^10^

### Immediate measures

To ensure the safety and security of their patients and staff:

- Rosters of staff cleared for duty must be organised;
- Large outbreaks may require re-allocation of units and hospital wards;
- Attendance at outpatients must be restricted or curtailed – those attending must have prior screening in a secured environment; crowding must be avoided and distancing ensured;
- Additional space for real or potential COVID-19 patients must be identified and made available;
- Numbers of ICU beds (and ventilators) available must be supplemented;
- The number of team members in specific departments (where they should remain) should be limited;
- Telemedicine, social media, Zoom online meetings for consultations, routine follow-up care, lectures, conferences, etc. must be fully utilised.

## Conclusion

To fulfil our duty, we must keep abreast of developments, adjust our practice appropriately, strengthen our ability and resilience in managing this infectious outbreak, but never neglect the need of patients who depend on us.
